# Impact of V9302, a Competitive Antagonist of Transmembrane Glutamine Flux on Reversal of Resistance in Breast Cancer Cell Lines

**DOI:** 10.3390/pharmaceutics16070877

**Published:** 2024-06-29

**Authors:** Nikoletta Szemerédi, Zsuzsanna Schelz, Dária Antónia Horvath, Bálint Rácz, András G. Szatmári, Hiba F. Muddather, Noémi Bózsity, István Zupkó, Gabriella Spengler

**Affiliations:** 1 Department of Medical Microbiology, Albert Szent-Györgyi Health Center and Albert Szent-Györgyi Medical School, University of Szeged, Semmelweis utca 6, 6725 Szeged, Hungary; szemeredi.nikoletta@med.u-szeged.hu (N.S.); balintracz95@gmail.com (B.R.);; 2 Institute of Pharmacodynamics and Biopharmacy, Faculty of Pharmacy, University of Szeged, Eötvös utca. 6, 6720 Szeged, Hungary; schelz.zsuzsanna@szte.hu (Z.S.); hiba.161991@hotmail.com (H.F.M.); bozsity-farago.noemi@szte.hu (N.B.)

**Keywords:** V9302, breast cancer, P-glycoprotein (ABCB1), cell cycle, apoptosis

## Abstract

Chemotherapy is a known treatment modality that improves the long-term survival of breast cancer patients. However, due to the resistance to numerous anticancer drugs, alternative chemotherapeutic strategies are required. Regarding antimetabolic drugs, several compounds have proven anticancer properties, such as statins. The present study aimed to investigate the in vitro effects of V9302, a competitive antagonist of glutamine flux, on different subtypes of breast cancers (estrogen, progesterone, and HER2 receptor-positive or negative, and Pgp-negative and Pgp-overexpressing). The interactions of V9302 with standard chemotherapeutic drugs (doxorubicin and cisplatin) were also determined by MTT staining on breast cancer cell lines. Furthermore, the influence of V9302 on the cell cycle of MCF-7 and its Pgp-overexpressing counterpart KCR was monitored by flow cytometry. It was shown that V9302 exerted synergistic interactions with doxorubicin in all breast cancer cell lines. In cell cycle analysis, the KCR cell line was more sensitive to V9302. After 48 h, cell proliferation was completely blocked, and elevated G1, suppressed S, and decreased G2/M could be detected. Inhibition of glutamate transport can be assumed to block resistance related to Pgp.

## 1. Introduction

Cancer is a well-known burden to the world that involves health care, social, and economic aspects, in addition to being more scientifically relevant to the pharmacology industry. According to WHO statistics, in 2022, more than 20 million new cancer cases and 9.7 million deaths were recorded [1]. Although the number of cancer patients worldwide is increasing, the number of FDA-approved anticancer drugs was only 14 in 2023 [2]. On the other hand, the number of approved drugs in 2023 was 55, almost four times that of anticancer drugs [3].

Drug repurposing, or drug repositioning, is the reuse of already approved-medications for purposes that they were initially not intended for [4]. Thus, this method enables researchers to discover new indications for approved drugs without the time-consuming and expensive drug development process. 

In general, drugs considered effective in cancer therapy are primarily cardiovascular drugs, antipsychotic drugs, antidiabetic drugs, antidepressants, microbiological agents, antiviral drugs, antibiotics, and NSAIDs [5]. Of the antimetabolic drugs, several compounds have proven anticancer properties, such as statins and metformin. Statins are prescribed for lipid disorders and can thus prevent cardiovascular diseases. They inhibit the HMG-CoA enzyme that catalyzes the initial step in cholesterol biosynthesis. This inhibition also disrupts cell cycle progression by inhibiting the mevalonate pathway and could have an anticancer effect [6]. These effects were confirmed by in vitro and in vivo studies [7]. However, until now, observational studies have not shown an apparent connection between cancer risk and statin use [8]. Metformin is a very effective antidiabetic drug in type 2 diabetes mellitus that inhibits hepatic gluconeogenesis and reduces glucose production. This drug suppresses metabolic glucose production by activating AMP-activated protein kinase in the liver [9]. It was suggested that the use of metformin in patients with type 2 diabetes decreases the incidence of several cancers, such as colorectal, lung, liver, breast, etc. [10]. It was confirmed that in breast cancer, metformin works as a growth-dependent inhibitor through AMP-kinase [11].

The present study aims to find drugs that act on cell metabolism that could have an anticancer effect on different breast cancer cell lines. In addition, based on previous experiments, the lead molecule of this study is V9302, an inhibitor of glutamine metabolism. The molecule V9302 is a competitive antagonist of transmembrane glutamine flux by potently and selectively targeting the amino acid transporter ASCT2. It hinders ASCT2-mediated glutamine uptake (IC_50_ = 9.6 µM) in HEK-293 cells [12,13]. ASCT2 (or SLC1A5), also known as an alanine–serine–cysteine transporter, is a Na-coupled transporter. Contrary to its name, its most important role is transporting glutamine. Glutamine transport plays a crucial role in aerobic glycolysis of breast cancer. Although there are some differences in the exact occurrence between the subtypes of breast cancer, ASCT2 is generally highly expressed in these cells [14]. Studies have been conducted on V9302 and its effect on glutamine transport. Li et al. found that V9302 in breast cancer cells promotes autophagy through the regulation of accumulation of reactive oxygen species (ROS), and combining V9302 with anti-PD-1 immunotherapy had a synergetic effect in mouse models [15]. A similarly constructed study, including a photodynamic immunostimulant consisting of BMS-1 (PD-1 inhibitor), V9302, and chlorin e6 (a photosensitizing agent), showed anti-tumor activity in metastases due to increased recognition between CD8+T cells and Fas-overexpressing tumor cells [16]. Furthermore, it was described that, in addition to the potential anticancer effect, V9302 suppresses osteoclast differentiation and, thus, may help prevent osteoporosis [17].

Among female malignancies, breast cancer has the highest incidence and mortality. The global incidence of breast cancer in 2022 is estimated to be 2.3 million new cases and 665 thousand deaths, representing 25% of new cancer cases and 1 in 6 cancer deaths among women [18]. Incidence rates vary approximately four times throughout the world, ranging from 26.2 per 100,000 in South Central Asia to 95.5 in Australia/New Zealand [19]. Differences in breast cancer incidence in different regions could be explained by differences in breast cancer risk factors, level of education achieved, average life expectancy in different areas, and screening programs [20]. There is no single identified cause for breast cancer, but there are many established risk factors that increase the chance that a female will develop breast cancer. These include the patient’s age [21], early menarche, late menopause [22], late age at first pregnancy [23], long-term use of hormone replacement therapy [24], and inherited mutation in the *BRCA1/BRCA2* gene [25]. Breast cancer could be classified into four primary molecular subtypes, defined by the presence or absence of hormone receptors (HR) and other types of proteins: (1) luminal A or HR+/HER2-; (2) luminal B or HR+/HER2+; (3) triple-negative or HR-/HER2-; and (4) HER2-positive [26]. These different subtypes vary markedly in incidence and prognosis [27]. Breast cancer management is a multimodal approach. Chemotherapy is a known treatment modality that improves long-term survival of breast cancer [28]. However, cancer cells exhibit intrinsic and acquired resistance to numerous anticancer drugs [29], a major obstacle to effectively treating cancer. Therefore, alternative chemotherapeutic strategies are required.

Presently, anthracycline and taxane-based therapy is applied for the treatment of breast cancer as cytotoxic therapy [30]. The therapeutic response, however, might be poor due to emerging drug resistance. Several resistance mechanisms are in the background of the ineffectiveness of chemotherapy, and P-glycoprotein-related resistance is a great burden. Intracellular drug concentrations are determined by the functions of different transporter proteins, and ATP-binding cassette (ABC)-related efflux mechanisms, such as P-glycoprotein (Pgp), play a substantial role in the multidrug resistance of cancers. Long-term treatment with a Pgp substrate antitumor agent increases the expression of the Pgp encoding gene [31]. Pgp (P-glycoprotein or ABCB1) is present in normal tissues, such as the liver, kidney, gastrointestinal tract, and blood–brain barrier; therefore, reversing multidrug resistance via blockade of Pgp holds a great challenge; therefore, cancer-selective approaches are principally expected. The overexpression of transporter proteins in cancers could provide some potential for higher selectivity for MDR-reversing agents. Verapamil was a promising candidate for antitumor combination therapy in the early era of research in the field of multidrug resistance reversal [32]. Unfortunately, clinical trials did not prove the efficacy of Pgp inhibitors, even third-generation drugs such as tariquidar [33]. Pgp-overexpressing breast cancer cells could be targeted by modulating solute carriers that could modulate the metabolic functions of cancer cells and could cause a disturbance of the energy supply of Pgp. 

As highlighted previously, our aim was to demonstrate the in vitro effects of V9302 on different subtypes of breast cancers, namely estrogen, progesterone, and HER2 receptor-negative ATCC HTB-26^TM^ (MDA-MB-231), estrogen receptor-positive, Pgp-overexpressing KCR, estrogen receptor-, and progesterone receptor-positive MCF-7 (ATCC HTB22 the parental non-resistant cell line of KCR), and estrogen receptor-positive T-47D (HTB-133^TM^). Previous research has shown a connection between Pgp overexpression and SLC7A11 (a cysteine transporter) inhibition [34]. We hypothesized that V9302 might reverse resistance in the Pgp-overexpressing KCR breast cancer cell line by inhibiting glutamine transport. To our knowledge, this is the first study to focus on the activity of V9302 in various breast cancer cell lines with different resistance patterns, including overexpressed Pgp-related resistance. Furthermore, the interaction of V9302 with standard chemotherapeutic drugs, such as doxorubicin and cisplatin, was also determined. To achieve our objectives, the antiproliferative and cytotoxic activities of the compounds and the combination studies were tested using MTT staining. Furthermore, the impacts of V9302 on the cell cycle of MCF-7 and its Pgp-overexpressing counterpart KCR were monitored by flow cytometry. To prove the activity of V9302 on the accumulation of the Pgp substrate rhodamine 123, the compound was tested on parental mouse T-lymphoma cells (PAR) and its Pgp-overexpressing counterpart (MDR) by flow cytometry. The results of the rhodamine 123 accumulation assay were also confirmed by the luminescent Pgp ATPase inhibition test.

## 2. Materials and Methods

### 2.1. Cell Lines and Their Maintenance

**Figure 1:**
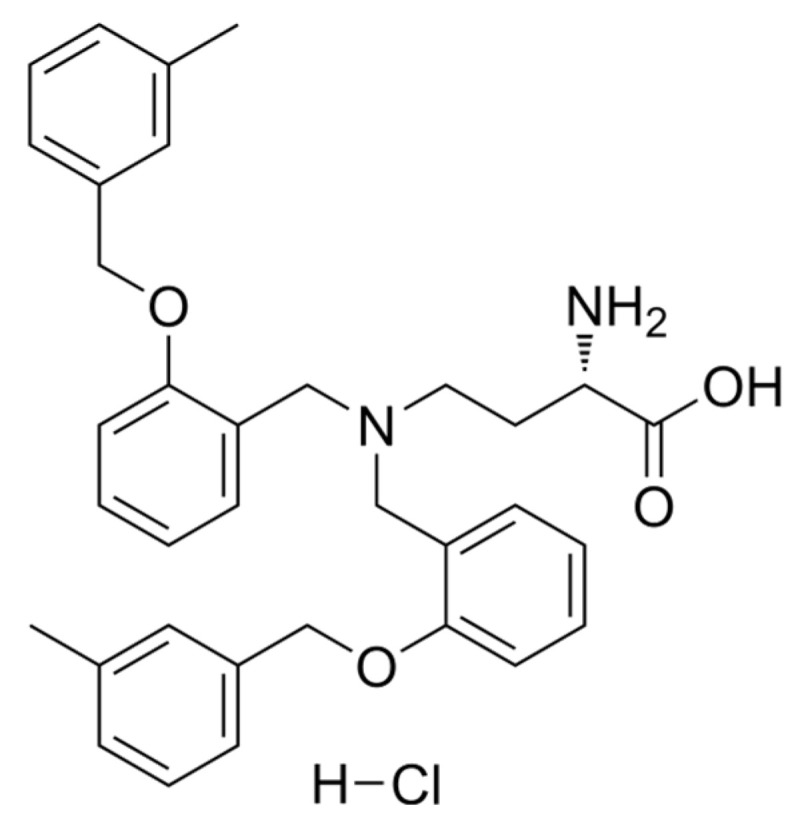
Chemical structure of V9302.

The effect of V9302 ((2S)-2-amino-4-[bis[[2-[(3-methylphenyl)methoxy]phenyl]methyl]amino]butanoic acid; MedChemExpress, Monmouth Junction, NJ, USA) (Figure 1) was investigated on several cell lines: breast cancer cell line MCF-7 (ATCC^®^ HTB-22) (purchased from LGC Promochem (Teddington, Middlesex, UK)), and its doxorubicin-resistant subline KCR; HTB-26™ breast adenocarcinoma (ATCC^®^ MDA-MB-231); and T-47D (ATCC^®^ HTB-133™) ductal carcinoma of the breast; L5178Y mouse T-lymphoma cells (ECACC catalog no. 87111908, US FDA, Silver Spring, MD, USA) were transfected with the pHa MDR1/A retrovirus, as previously reported [35]. The ABCB1 (P-gp)-overexpressing cells were selected by culturing the infected cells with 60 ng/mL of colchicine (Sigma-Aldrich Chemie GmbH, Steinheim, Germany) to preserve the MDR phenotype. Cells were grown in Eagle’s minimal essential medium (EMEM), containing 4.5 g/L of glucose supplemented with a non-essential amino acid (NEAA) mixture (Sigma-Aldrich, St Louis, MO, USA), a selection of vitamins, and 10% heat-inactivated fetal bovine serum (FBS) (Biosera; Cholet, France). L5178Y (parental, PAR) mouse T-cell lymphoma cells and the human ABCB1-transfected subline were cultured in McCoy’s 5A supplemented with 10% heat-inactivated horse serum, 100 U/L L-glutamine, and 100 mg/L penicillin–streptomycin mixture, all obtained from Sigma-Aldrich. Cell lines were incubated at 37 °C, in an atmosphere of 5% CO_2_ and 95% air. In the case of KCR, on every third passage, 0.56 µg/mL doxorubicin (Teva Pharmaceuticals; Tel-Aviv, Israel) was added to the medium in order to maintain ABCB1 expression.

### 2.2. Antiproliferative and Cytotoxicity Assays

The antiproliferative and cytotoxic effects of the compounds (doxorubicin, cisplatin, V9302) were determined on the breast cell lines mentioned above. The cytotoxic activity of V9302 was determined in mouse lymphoma cell lines (PAR and MDR). The effects of increasing concentrations of the compound were tested on 96-well flat bottom microtiter plates. V9302 was diluted in 200 μL of medium. The density of the cells was adjusted to 6 × 10^3^ cells (antiproliferative assay) or 1 × 10^4^ cells (cytotoxicity assay) in 100 μL per well. The cells were seeded for 24 h at 37 °C with 5% CO_2_ prior to the assay, then the medium was removed from the plates containing the cells and 100 μL of fresh medium was added. The 10 mM stock solution of V9302 was prepared in dimethylsulfoxide (DMSO; Sigma-Aldrich, St Louis, MO, USA). The dilutions of the compounds were previously made in a separate plate and added to the cells in 100 μL. The starting concentration of cisplatin and V9302 was 100 μM; in the case of doxorubicin, it was 8.62 μM, and then a two-fold serial dilution was performed (concentration range: 100–0.19 μM or 8.62–0.02 μM, respectively). Culture plates were incubated at 37 °C for 72 h (antiproliferative test) or 24 h (cytotoxicity test) at the end of the incubation period, 20 μL of thiazolyl blue tetrazolium bromide (MTT; Sigma-Aldrich, St Louis, MO, USA) solution (from a stock solution of 5 mg/mL) was added to each well. After incubation at 37 °C for 4 h, 100 μL of sodium dodecyl sulfate (SDS; Sigma-Aldrich, St Louis, MO, USA) solution (10% in 0.01 M HCI) was added to each well, and the plates were further incubated at 37 °C overnight. Cell growth was determined by measuring optical density (OD) at 540/630 nm with a Multiscan EX ELISA reader (Thermo Labsystems, Cheshire, WA, USA). The IC_50_ values and standard deviation (SD) of triplicate experiments were calculated using GraphPad Prism software, version 5.00 for Windows, with a nonlinear regression curve fit. DMSO was used as a solvent control; cisplatin and doxorubicin were used as positive controls. The results are expressed in terms of IC_50_, defined as the inhibitory dose that reduces the proliferation of cells exposed to the compounds tested by 50%. DMSO was used as a solvent control; cisplatin (CIS) and doxorubicin (DOX) were used as positive controls.

### 2.3. Combination Assay

The checkerboard microplate method was applied to study the effect of drug interactions between V9302 and the reference chemotherapy drugs doxorubicin (DOX) and cisplatin (CIS) in the different breast cancer cell lines mentioned above. DOX and CIS dilutions were made in the direction from left to right in 100 μL (final concentrations used in the assay are presented in Table 3), and V9302 dilutions were made from top to bottom in the microtiter plate in 50 μL volume. The concentrations of V9302 were determined based on the antiproliferative results. Cells were seeded for 24 h at 37 °C with 5% CO_2_, then the medium was removed from the plates, 50 μL of fresh medium was added to the cells, and the number of cells was adjusted to 6 × 10^3^ cells per well. The plates were incubated for 72 h. At the end of the incubation period, the cell growth rate was determined by the MTT assay described above. The combination index (CI) values at 50% of the growth inhibition dose (ED_50_) were determined using CalcuSyn software to plot four to five data points in each ratio. CI values were calculated using the median effect equation according to the Chou–Talalay method (Table 1), where CI < 1, CI = 1, and CI > 1 represent synergism, additive effect (or no interaction), and antagonism, respectively [36].

### 2.4. Cell Cycle Analysis

Cell cycle analysis was performed to measure the cellular DNA content of the cells using flow cytometry. Briefly, estrogen and progesterone receptor-positive MCF-7 and doxorubicin-resistant KCR cells were seeded into 12-well plates at a density of 200,000 cells/well and incubated overnight. The cells were treated with increasing concentrations of the test compounds for 24 h and 48 h. The cells were then washed with phosphate-buffered saline (PBS; Capricorn Scientific GmbH, Ebsdorfergrund, Germany), collected after trypsinization, and centrifuged at 1400 rpm for 5 min at room temperature. After carefully removing the supernatants, cells were washed and fixed in 300 µL of 70% ice-cold ethanol for 30 min. Finally, cells were stained with a propidium iodide (PI) solution (10 µg/mL PI, 10 µg/mL RNase A, 0.1% Triton-X, and 0.1% sodium citrate dissolved in distilled water; PI and Triton-X; Sigma-Aldrich, St Louis, MO, USA) and incubated in the dark for 30 min at room temperature. Detection of the DNA content from at least 20,000 events was carried out with a CytoFlex flow cytometer (Beckman Coulter, Brea, CA, USA). The recorded data (histograms) were analyzed using ModFit LT 3.3.11 software (Verity Software House, Topsham, ME, USA) to determine the percentages of cells in the different phases of the cell cycle. Untreated cells were considered controls. Hypodiploid cells (subG1) were regarded apoptotic cells. The column diagrams contain the results of two independent experiments with triplicates, with statistical analysis by GraphPad Prism10.

### 2.5. Rhodamine 123 Accumulation Assay

Rhodamine 123 (R123) is a non-toxic, lipophilic, cationic fluorescent dye (λex/em = 505/534 nm), which is a substrate of the ABCB1 transporter. As the compound is membrane-permeable, it is rapidly taken up by the cells, therefore, it can be effectively used for the screening of efflux pump-inhibiting compounds. In the parental cell line (PAR), high intracellular fluorescence occurs, while its Pgp-overexpressing counterpart (MDR) has low fluorescence. The treated samples were compared with the fluorescence of PAR cells that was considered 100%. V9302 was tested at 2, 10, and 20 µM. Tariquidar (positive control, Sigma-Aldrich, St. Louis, MO, USA) was used at 0.2 µM. The fluorescence of the gated cell population was measured with the CytoFlex flow cytometer (Beckman Coulter, Brea, CA, USA) [37]. 

### 2.6. Pgp ATPase Assay

The P-glycoprotein ATPase activity was measured using the Pgp-Glo^TM^ Assay Systems (Promega) kit following the manufacturer’s protocol. V9302 was tested at a 20 µM concentration because this concentration produced a significant result in the rhodamine 123 accumulation assay. Sodium orthovanadate (Na_3_VO_4_, 0.25 mM) was used as an inhibitor control, and verapamil (0.5 mM) served as a substrate control. The luminescent signal generated by luciferase was measured using a CLARIOstar Plus plate reader (BMG Labtech, UK) at 580 nm [37].

## 3. Results

### 3.1. Cytotoxic and Antiproliferative Activities

Based on the results, V9302 was shown to be cytotoxic in MCF-7 and MDA-MB-231 cell lines (IC_50_: 4.68 and 19.19 μM, respectively); a weaker effect was observed in T-47D cells, and V9302 did not show cytotoxic activity in KCR cells (Table 2). However, in the case of the antiproliferative assay, where low cell number and 72 h incubation time were applied, V9302 was effective in cell proliferation in all cell lines; the most potent effect was presented in the case of the MCF-7 cell line (IC_50_: 2.73 μM) (Table 2). Except for KCR, all cell lines were susceptible to doxorubicin; however, cisplatin showed less activity (Table 2). The cytotoxic activity of V9302 was also assessed on PAR (IC_50_ = 11.55 ± 0.44 μM) and MDR (IC_50_ = 14.2 ± 0.91 μM) cells.

### 3.2. Combination Assay

A microplate checkerboard technique was used to investigate the impact of drug interactions involving V9302 in conjunction with chemotherapeutic agents, doxorubicin, and cisplatin. The results were presented as combinations index (CI) values at 50% growth inhibition (ED_50_), derived by plotting 4 or 5 data points for each ratio using CalcuSyn software. This software facilitates the computation of combination indexes and identifies the most powerful ratios of the combined agents. Synergistic interactions between V9302 and doxorubicin and cisplatin were observed in several breast cancer cell lines. The evaluation of interaction types in combination studies used the Chou–Talalay method, rooted in the median effect equation. With respect to this calculation, the dose–effect curves for each drug alone are essential. This method allows the testing of multiple ratios of drug combinations, enabling a more accurate characterization of interaction types depending on the concentrations of the compounds [38,39].

V9302 showed synergism with doxorubicin and cisplatin in certain ratios in all breast cancer cell lines tested (Table 3); however, in the case of MCF-7 cells, all combination ratios tested for V9302 and cisplatin showed antagonism (see Supplementary Material, Table S1). In addition, other interaction types, such as additive effects and antagonism, were also observed depending on the ratio of compounds in the assays (see Supplementary Material).

In the case of the KCR cell line, another approach was used to determine the interaction of V9302 and doxorubicin. The KCR cell line is resistant to doxorubicin, and high concentrations of doxorubicin should have been applied in the checkerboard method; however, MTT staining interferes with the intensive color of doxorubicin. For this reason, since doxorubicin did not have an IC_50_ in KCR cells (IC_50_ > 8.62 μM), we used doxorubicin at a double concentration (17.24 μM) that still has no effect on cell viability and whose color does not interfere with MTT to check the change in the IC_50_ value of doxorubicin. V9302 was applied at IC_50_ (24.45 μM) or IC_50_/2 (12.22 μM) at a constant concentration with serially diluted two-fold doxorubicin concentrations (starting concentration of doxorubicin: 17.24 μM) in triplicates. Plates were prepared according to the parameters described in the combination assay (cell number, incubation time) (Table 4). It was shown that DOX alone had no effect on KCR cells, but when applied together with V9302 at the concentration of IC_50_, the IC_50_ of doxorubicin could not be determined because this combination was very toxic to the cells, confirming the synergistic interaction between V9302 and doxorubicin. Furthermore, a strong synergistic interaction was observed at the IC_50_/2 of V9302 together with doxorubicin: the IC_50_ of doxorubicin decreased to 0.685 μM.

### 3.3. Cell Cycle Analysis

**Figure 2:**
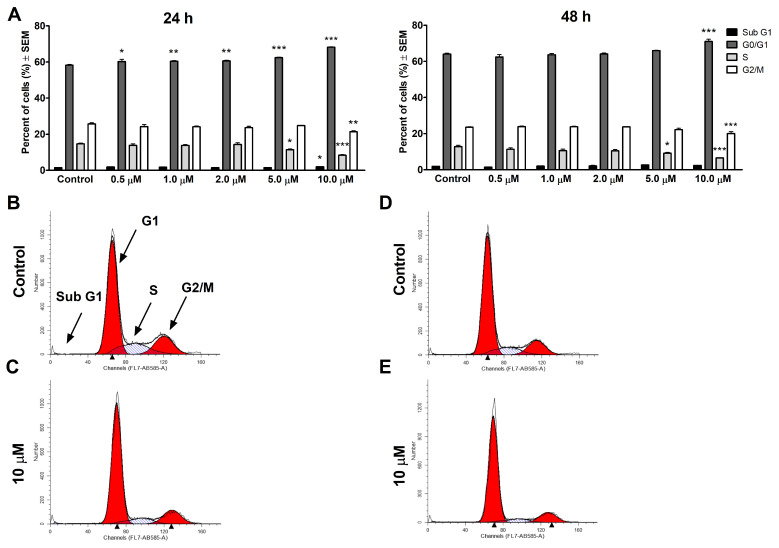
V9302 causes changes in the cell cycle distribution in MCF-7 cells. The figure represents the percentages of the different cell cycle phases after the 24 and 48 h treatment with V9302. Data presented are mean ± SEM, *, ** and *** indicate *p* < 0.05, *p* < 0.01 and *p* < 0.001, respectively, compared to control samples (**A**). Selected histograms exemplify the cell cycle disturbances caused by V9302 at different concentrations after 24 h (**B**,**C**) and 48 h (**D**,**E**).

**Figure 3:**
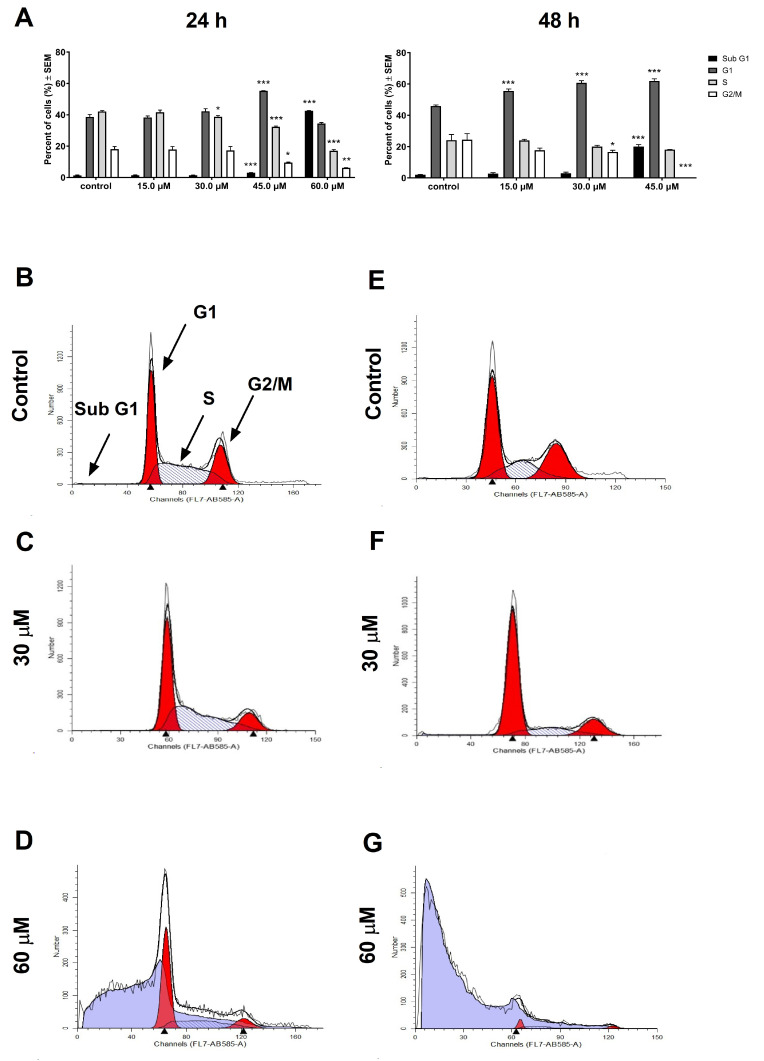
V9302 effects on the cell cycle distribution in Pgp-overexpressing KCR cells. Bar charts represent the percentages of the different cell cycle phases after the 24 and 48 h treatment with V9302. Data presented are mean ± SEM, *, ** and *** indicate *p* < 0.05, *p* < 0.01 and *p* < 0.001, respectively, compared to control samples (**A**). Selected histograms exemplify the cell cycle disturbances caused by V9302 at different concentrations after 24 h (**B**–**D**) and 48 h (**E**–**G**).

The impact of V9302 on cell cycle progression was investigated in vitro at 24 and 48 h through the analysis of the cellular DNA content using a flow cytometer. Analysis was carried out on two selected breast cancer cell lines representing luminal A conditions with a doxorubicin-sensitive (MCF-7) and a doxorubicin-resistant (KCR) cell type (Figure 2 and Figure 3). The applied concentrations of V9302 were selected based on the cytotoxicity and antiproliferative assay results, and a consistently growing amount of the compound was added to the cell cultures. In the case of MCF-7, the applied concentrations could be increased up to 10 µM, which means a twofold increase compared to the cytotoxicity IC_50_ value (4.68 ± 0.07 µM), and there was a relatively low rate but significant cell cycle disturbance. After 24 h of incubation, a substantial accumulation of the G1 phase and a concentration-dependent decrease of the S phase could be observed (Figure 2). At a concentration of 10 µM, a significant decline was also detected in the G2/M phase. However, the subG1 phase that corresponds to the apoptotic population did not change substantially. A longer incubation of 48 h resulted in significant cell cycle disturbances only at the two highest concentrations tested, characterized by G1 phase accumulation and reduction at the S and G2/M phases at a concentration of 10 µM, with a minimal reduction in the S phase at a lower 5 µM concentration (Figure 2).

KCR cells were sensitive to the compound under the cytotoxic and antiproliferative IC_50_ values as well. The highest applied concentration induced substantial cell cycle disturbance and toxicity under the present conditions. After 24 h, the 60 µM test compound resulted in massive apoptosis in the entire cell population, and after 48 h, cell proliferation was completely blocked and a negligible number of cells could be collected (Figure 3). Elevated G1, suppressed S, and decreased G2/M could be detected in the resistant cell line. The subG1 population increased significantly in 45 µM concentration after 24 and 48 h (Figure 3). 

### 3.4. Rhodamine 123 Accumulation Assay

**Figure 4:**
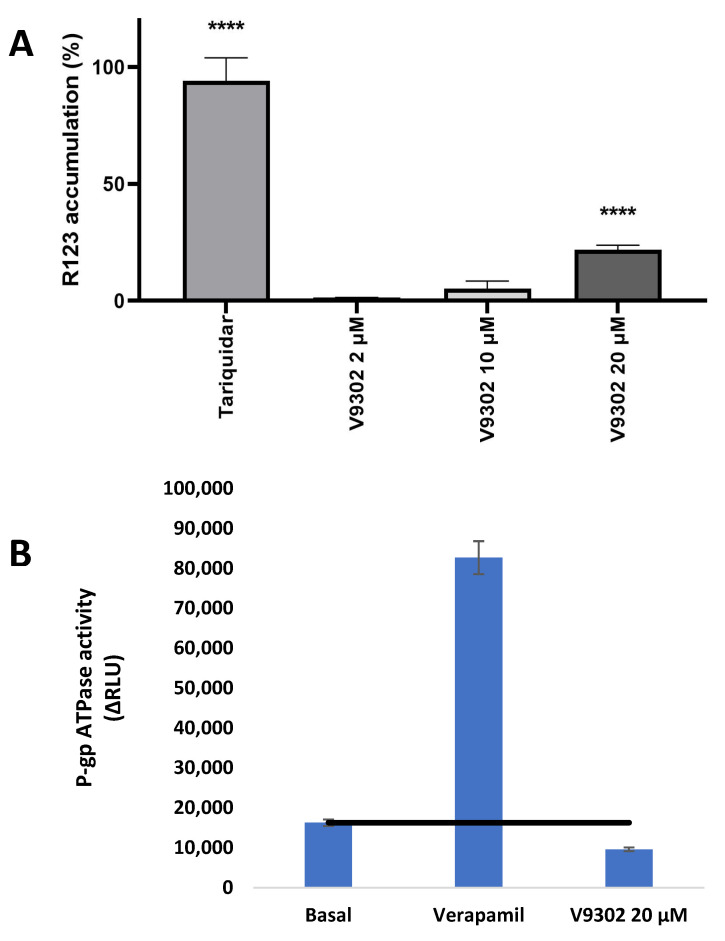
(**A**): Rhodamine 123 accumulation in Pgp-overexpressing mouse T-lymphoma cells in the presence of V9302 at 2, 10, and 20 µM (**A**). Tariquidar was used at 0.2 µM as a positive control. PAR cells represent 100% rhodamine 123 accumulation. (**B**): Pgp ATPase inhibition: V9302 was applied at 20 µM. ∆RLU_TC_ > ∆RLU_basal_: the tested compound (TC) is stimulator of Pgp ATPase activity; ∆RLU_TC_ = ∆RLUbasal: the tested compound has no effect on Pgp ATPase activity; ∆RLU_TC_ < ∆RLU_basal_: the tested compound is an inhibitor of Pgp ATPase activity, black line represents the basal Pgp ATPase activity, **** indicates *p* < 0.0001.

The ability of compounds to inhibit Pgp efflux activity was evaluated using the rhodamine 123 functional assay by flow cytometry on a sensitive mouse T-lymphoma cell line (L5178Y-PAR) and its human *MDR1*-transfected MDR subline (L5178Y-MDR) [37]. The fluorescence of intracellular rhodamine 123 accumulation was measured in resistant, Pgp-overexpressing MDR and in sensitive PAR cancer cells. V9302 was tested at concentrations of 2, 10, and 20 µM (Figure 4A). The treated samples were compared with the fluorescence of PAR cells that was considered 100%. It was confirmed that V9302 significantly inhibited Pgp in MDR cells at 20 µM (Figure 4A).

### 3.5. Pgp ATPase Assay

The ATPase activity assay was based on the ATP-dependent light-generating reaction of firefly luciferase. After incubation of Pgp with ATP, the reaction was halted, and the ATP consumption by Pgp was indicated by a luminescent signal produced by the remaining unmetabolized ATP. The relative ATPase activity was determined by calculating the ratio between the luminescence measured for the Pgp ATPase activity of the compound V9302 and the basal P-gp ATPase activity. According to the results, V9302 was demonstrated to be an inhibitor of Pgp (Figure 4B).

## 4. Discussion

The complexity of cancer, marked by its diverse nature, rapid acquisition of resistance to treatment, and the intricate network of cellular pathways that drive its aggressiveness, poses a significant obstacle for single-agent treatments. It is becoming increasingly clear that the most promising path forward in cancer treatment involves the strategic combination of therapies [40]. Cancer is a dynamic, multifaceted condition characterized by disruptions in numerous cellular systems (DNA repair, apoptosis, immune response, and metabolism). As tumor cells multiply, they undergo metabolic pathway reprogramming; furthermore, various oncogenes and tumor suppressors have been implicated in cancer-related alterations in metabolism. This suggests that early-stage cancer might be identifiable by monitoring simple metabolic alterations. Consequently, cancer could be described as a metabolic disease [41]. For this reason, compounds affecting metabolic pathways could be used as single agents or in combination therapy against cancer.

In the present study, the anticancer potential of V9302, an inhibitor of glutamine metabolism, was investigated alone and in combination with standard chemotherapeutic drugs on breast cancer cell lines with different resistance patterns. Previously, it was confirmed that V9302 interferes with the cell cycle, induces apoptosis, and generates reactive oxygen species (ROS) [13]. Furthermore, V9302 induced autophagy in breast cancer cells, and synergistic activity between anti-PD-1 and V9302 was observed [15].

Our study aimed to investigate the role of Pgp in breast cancer and the activity of V9302 on MCF-7 and its Pgp-overexpressing counterpart KCR. Doxorubicin-resistant KCR also shows resistance to the compound V9302, however, this ASCT2 inhibitor has relevant toxicity in the doxorubicin-sensitive counterpart. There are approximately 20-fold higher IC_50_ values in cytotoxicity and antiproliferative assays for KCR cells. To clarify the possible mechanisms, a cell cycle analysis was performed by flow cytometry. Cell cycle analysis was performed by flow cytometry, providing additional information on the possible mechanism of action of V9302. MCF-7 cells treated with the compound exhibited minimal but significant cell cycle disturbance features. After 24 h of incubation, a concentration-dependent increase was observed in the G1 phase accompanied by a significant decrease in the S phase and G/M phases at a concentration of 10 µM. However, extended incubation periods of 48 h resulted in significant changes in cell cycle distribution only at the highest 10 µM concentration. On the contrary, doxorubicin-resistant KCR cells were more sensitive to V9302 compared to MCF-7 cells. Specifically, treated cells exhibited cell cycle depression in the S and G2/M phases, accompanied by a marked increase in the hypodiploid subG1 population, particularly at the concentration of 60 µM after 24 h of incubation. Forty-eight hours after treatment of KCR cells, a concentration- and time-dependent elevation of the G1 phase and a reduction of the S and G2/M phases, with a significant increase in the hypodiploid subG1 population, were observed. It is noteworthy that at 60 µM concentration, cell proliferation was completely inhibited, and barely any KCR cells could be collected at this time interval. The withdrawal of glutamate by inhibiting uptake mechanisms was confirmed to also be a beneficial approach to combating resistance related to Pgp, since Pgp expression could be driven by a higher concentration of glutamate [42]. V9302 is an inhibitor of Pgp and could reverse the resistance related to Pgp of MDR mouse T-lymphoma cells. A similar phenomenon was observed in KCR cells that were more sensitive to V9302 compared to MCF-7 cells, since after 48 h posttreatment, cell proliferation was completely blocked, the G1 phase was accumulated, the S phase was suppressed, and the G2/M phase was reduced. Apoptosis was induced by a concentration of V9302 that was below the antiproliferative IC_50_ value.

Regarding the combination studies, V9302 exerted synergistic interactions with doxorubicin in all breast cancer cell lines. Interestingly, all combinations of V9302 and cisplatin tested were antagonistic in MCF-7 cells, possibly due to their target molecules in MCF-7 cells. It can be assumed that V9302 can modulate the activity of Pgp and, consequently, doxorubicin, a well-known Pgp substrate, could achieve a higher intracellular concentration in KCR cells when applied together with V9302. Furthermore, the types of interactions between V9302 and doxorubicin or cisplatin depend on the ratios used in the studies. This important finding highlights the role of personalized parameters (e.g., best combination ratio, type of resistance) in the treatment of breast cancers.

## 5. Conclusions

The withdrawal of glutamate by inhibiting the uptake mechanisms seems to be a beneficial approach to combating resistance related to Pgp. V9302, an inhibitor of glutamine metabolism, affects the cancer MDR efflux pump Pgp by inhibiting the ATPase activity of the pump. KCR, the breast cancer cell line that overexpresses Pgp, is more sensitive to V9302 compared to MCF-7 cells, as after 48 h post-treatment, cell proliferation was completely blocked and apoptosis was induced. The chemotherapeutic drug doxorubicin, a well-known Pgp substrate, could achieve a higher intracellular concentration in Pgp-overexpressing KCR cells when applied together with V9302, highlighting the synergistic nature of this drug combination. There is plenty of evidence that inhibition of glutamate transport in various types of cancer enhances the efficacy of tumor therapy. V9302 seems to be a good candidate for the pharmacotherapy of malignancies that can be targeted at the metabolic level.

## Figures and Tables

**Table 1:** Type of interactions based on combination indexes (CI) [36].

CI	Interaction	CI	Interaction
0–0.1	very strong synergism	0.9–1.1	additive effect
0.1–0.3	strong synergism	1.1–1.2	slight antagonism
0.3–0.7	synergism	1.2–1.45	moderate antagonism
		1.45–3.3	antagonism
0.7–0.85	moderate synergism	3.3–10	strong antagonism
0.85–0.9	slight synergism	>10	very strong antagonism

**Table 2:** Cytotoxic and antiproliferative effects of V9302, doxorubicin, and cisplatin on various breast cancer cell lines.

	MCF 7				MDA-MB-231				T-47D				KCR			
	CT		AP		CT		AP		CT		AP		CT		AP	
	IC_50_	SD	IC_50_	SD	IC_50_	SD	IC_50_	SD	IC_50_	SD	IC_50_	SD	IC_50_	SD	IC_50_	SD
V9302	4.68	0.21	2.73	0.07	19.19	1.11	21.88	1.18	41.11	1.26	18.24	1.13	>100	-	24.45	0.27
DOX	2.14	0.57	0.03	0	N/A		2.35	0.07	N/A		1.42	0.24	>8.62	-	>8.62	-
CIS	20.63	1.89	4.93	0.49	>100	-	39.19	0.72	>100	-	89.95	1.09	>100	-	2.21	0.36
DMSO	>2%	-	>2%	-	>2%	-	>2%	-	>2%	-	>2%	-	>2%	-	>2%	-

CT: cytotoxic, AP: antiproliferative, DOX: doxorubicin, CIS: cisplatin, DMSO: dimethyl-sulfoxide, SD: standard deviation.

**Table 3:** Synergistic interactions of V9302 with doxorubicin and cisplatin in breast cancer cell lines. Ratio *: the applied combination and the concentration of V9302:DOX or V9302:CIS combination.

Cell Line	Compound	Chemotherapeutic Drug	Starting Concentration (µM)	Ratio *	Combination Index (CI)	SD ±	Type of Interaction at ED_50_
MDA-MB-231	V9302	CIS	90 µM	0.9:1	0.87	0.063	slight synergism
				1.8:1	0.72	0.166	moderate synergism
		DOX		20.88:1	0.82	0.014	moderate synergism
				41.76:1	0.74	0.136	moderate synergism
				83.52:1	0.83	0.525	moderate synergism
				167.05:1	0.13	0.022	strong synergism
T47D	V9302	CIS	80 µM	0.8:1	0.5	0.06	synergism
				1.6:1	0.87	0.17	slight synergism
				3.2:1	0.66	0.12	synergism
				6.4:1	0.78	0.07	moderate synergism
		DOX		37.12:1	0.73	0.11	moderate synergism
KCR	V9302	CIS	100 µM	4:1	0.89	0.02	slight synergism
				8:1	0.79	0.03	moderate synergism
				32:1	0.88	2.21	slight synergism
MCF-7	V9302	DOX	10.92 µM	1.27:1	0.31	0.37	synergism
				2.53:1	0.88	0.19	slight synergism
				5.07:1	0.84	0.12	moderate synergism
				10.13:1	0.67	0.092	synergism
				20.27:1	0.86	0.1	moderate synergism

DOX: doxorubicin, CIS: cisplatin, SD: standard deviation, ED_50_: 50% growth inhibition.

**Table 4:** Interaction of V9302 and doxorubicin in the KCR breast cancer cell line.

	IC_50_ (μM) of Doxorubicin	SD ±
V9302 IC_50_ + DOX	<0.033	-
V9302 IC_50_/2 + DOX	0.685	0.050
DOX	>17.24	-

## Data Availability

The raw data supporting the conclusions of this article will be made available by the authors on request.

## Supplementary Materials

The following supporting information can be downloaded at: https://www.mdpi.com/article/10.3390/pharmaceutics16070877/s1, Table S1: Interactions of V9302 with doxorubicin and cisplatin in breast cancer cell lines (HTB-26, T47D, KCR, MCF-7). Figure S1A: Photo of the combination plate of doxorubicin and V9302 on MDA-MB-231 cells. B: Photo of the combination plate of cisplatin and V9302 on MDA-MB-231. Figure S2A: Photo of the combination plate of doxorubicin and V9302 on T47D cells. B: Photo of the combination plate of cisplatin and V9302 on T47D cells. Figure S3A: Photo of the combination plate of doxorubicin and V9302 on KCR cells. B: Photo of the combination plate of cisplatin and V9302 on KCR cells. Figure S4A: Photo of the combination plate of doxorubicin and V9302 (at concentration IC50 and IC50/2 on KCR cells. Figure S5A: Photo of the combination plate of cisplatin and on MCF-7 cells. Figure S6: Graphs of checkerboard combination assay on MDA-MB-231 cells with doxorubicin and V9302 at ratio 167.05:1. Figure S7: Graphs of checkerboard combination assay on MDA-MB-231 cells with cisplatin and V9302 at ratio 0.9:1. Figure S8: Graphs of checkerboard combination assay on T47D cells with doxorubicin and V9302 at ratio 37.12:1. Figure S9. Graphs of checkerboard combination assay on T47D cells with cisplatin and V9302 at ratio 0.8:1. Figure S10: Graphs of checkerboard combination assay on KCR cells with cisplatin and V9302 at ratio 4:1. Figure S11: Graphs of checkerboard combination assay on MCF-7 cells with doxorubicin and V9302 at ratio 20.27:1. Figure S12: Rhodamine-123 accumulation by parental (PAR) L5178Y mouse T-lymphoma and its Pgp overexpressing counterpart (MDR). Tariquidar was used at 0.2 µM as a positive control on MDR cells, V9302 was applied at 20 µM on MDR cells.
