# Supplementary material for: Impact of V9302, a Competitive Antagonist of Transmembrane Glutamine Flux on Reversal of Resistance in Breast Cancer Cell Lines
Source: Pharmaceutics. 2024 Jun 29;16(7):877. doi: 10.3390/pharmaceutics16070877 (PMC11280048; doi:10.3390/pharmaceutics16070877)
Supplement: Supplementary file 1 [file pharmaceutics-16-00877-s001.zip › pharmaceutics-3006283-supplementary.pdf]

Supplementary information

Impact of V9302, a competitive antagonist of transmembrane glutamine flux on resistance reversal in breast cancer cell lines

Nikoletta Szemerédi[1§], Zsuzsanna Schelz[2§], Dária Antónia Horvath[1], Bálint Rácz[1], András Gyula Szatmári[1], Hiba F. Muddather[2], Noémi Bózsity-Faragó[2], István Zupkó[2*], and Gabriella Spengler[1*]

[1] Department of Medical Microbiology, Albert Szent-Györgyi Health Center and Albert Szent-Györgyi Medical School, University of Szeged, Semmelweis utca 6, 6725 Szeged, Hungary; szemeredi.nikoletta@med.u-szeged.hu (N.S.); balintracz95@gmail.com (B.R.)

2 Institute of Pharmacodynamics and Biopharmacy, Faculty of Pharmacy, University of Szeged, Eötvös u. 6, H-6720 Szeged, Hungary; schelz.zsuzsanna@szte.hu (Z.S.); hiba.161991@hotmail.com (H.F.M.); bozsity-farago.noemi@szte.hu (B.N.)

* Correspondence: zupko.istvan@szte.hu (I.Z.); spengler.gabriella@med.u-szeged.hu (G.S.)

§ These authors contributed equally to this work.

Table S1. Interactions of V9302 with doxorubicin and cisplatin on breast cancer cell lines (HTB-26, T47D, KCR, MCF-7).

Cell lines	Compounds	Starting concentration of V9302 (μM)	Ratio	Combination index	SD ±	Type of interaction
MDA-MB-231	V9302+CIS	90	0.9:1	0.871	0.06	slight synergism
			1.8:1	0.722	0.17	moderate synergism
			3.6:1	1.272	0.42	moderate antagonism
			7.2:1	103.686	28.89	very strong antagonism
			14.4:1	84.075	31.77	very strong antagonism
			28.8:1	142.592	28.77	very strong antagonism
	V9302+DOX	90	10.44:1	1.231	0.34	moderate antagonism
			20.88:1	0.819	0.014	moderate synergism
			41.76:1	0.747	0.14	moderate synergism
			83.52:1	0.828	0.53	moderate synergism
			167.05:1	0.133	0.02	strong synergism
T-47D	V9302+CIS	80	0.8:1	0.579	0.06	synergism
			1.6:1	0.872	0.17	slight synergism
			3.2:1	0.664	0.12	synergism
			6.4:1	0.785	0.07	moderate synergism
			12.8:1	35.675	15.28	very strong antagonism
			25.6:1	10.901	4.08	very strong antagonism
	V9302+DOX	80	9.28:1	1.202	0.11	moderate antagonism
			18.56:1	61.619	23.83	very strong antagonism
			37.12:1	0.732	0.11	moderate synergism
			74.24:1	1.157	0.21	slight antagonism
			148.48:1	22.130	5.53	very strong antagonism
			296.96:1	17.848	7.16	very strong antagonism
KCR	V9302+CIS	100	1:01	58.030	8.21	very strong antagonism
			2:01	0.967	0.06	additive effect
			4:01	0.886	0.02	slight synergism
			8:01	0.787	0.03	moderate synergism
			16:01	1.551	0.27	antagonism
			32:1.00	0.884	2.21	slight synergism
MCF-7	V9302+CIS	10.92	0.11:1	1.46	0.18	antagonism
			0.22:1	1.515	0.188	antagonism
			0.44:1	1.667	0.17	antagonism
			0.87:1	1.742	0.25	antagonism
			1.75:1	1.741	0.36	antagonism
			3.5:1	1.608	0.3	antagonism
	V9302+DOX	10.92	1.27:1	0.314	0.37	synergism
			2.54:1	0.884	0.19	slight synergism
			5.067:1	0.84	0.12	moderate synergism
			10.13:1	0.672	0.09	synergism
			20.27:1	0.864	0.1	moderate synergism
			40.54:1	1.257	0.19	moderate antagonism

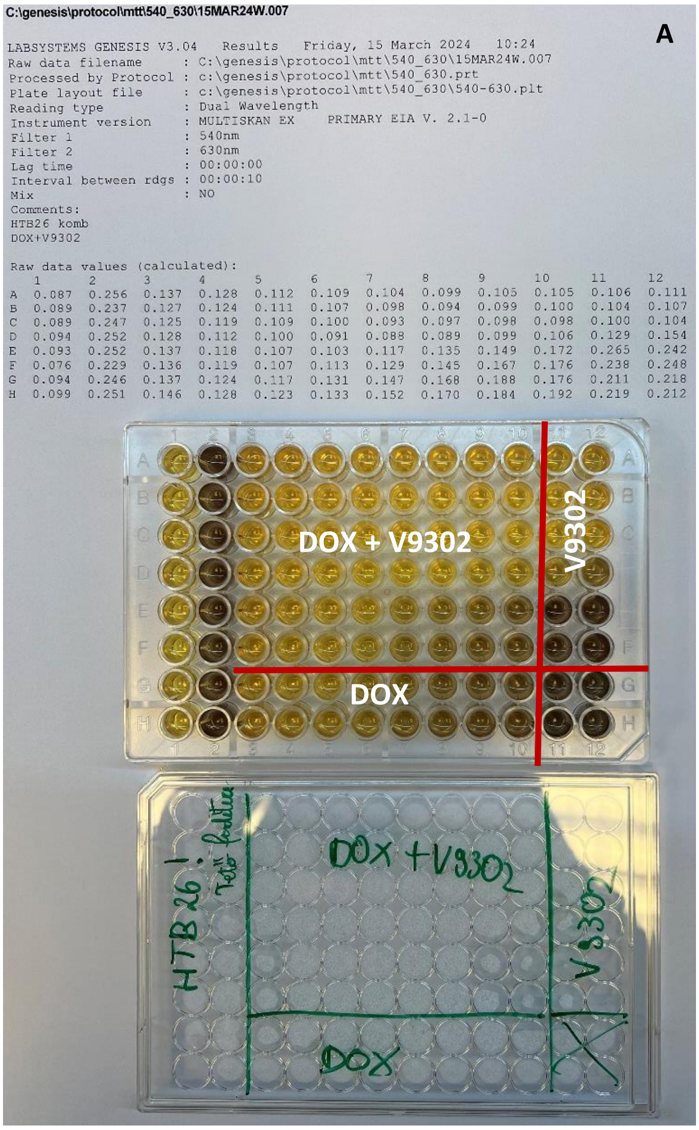

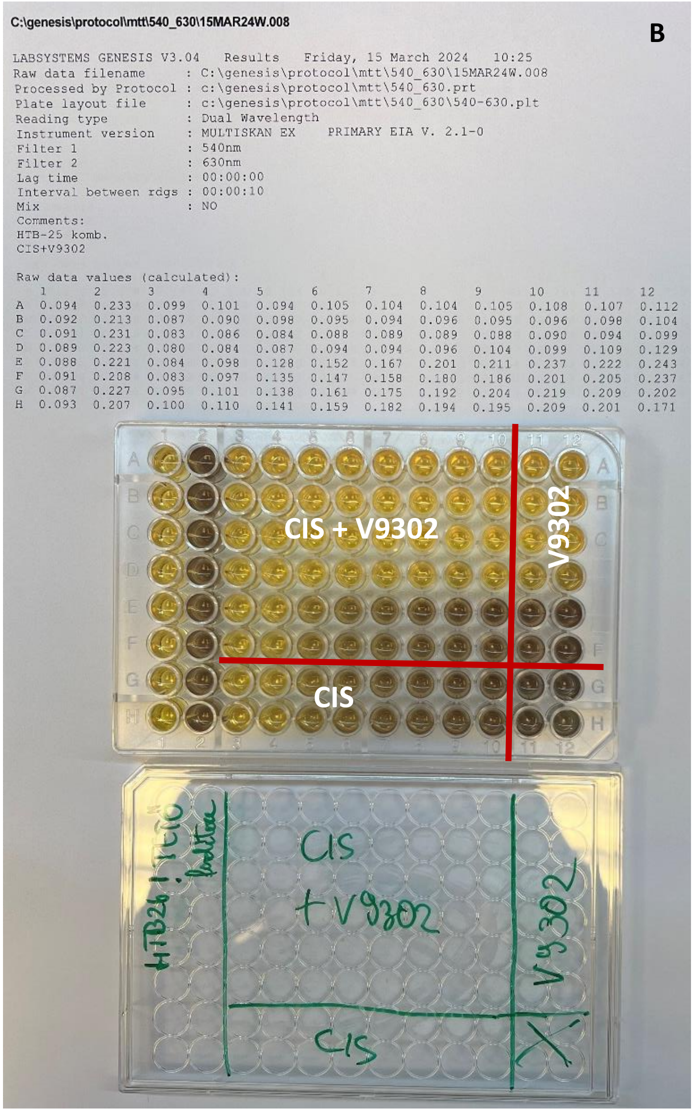

Figure S1A: Photo of the combination plate of doxorubicin and V9302 on MDA-MB-231 cells. **B:** Photo of the combination plate of cisplatin and V9302 on MDA-MB-231.

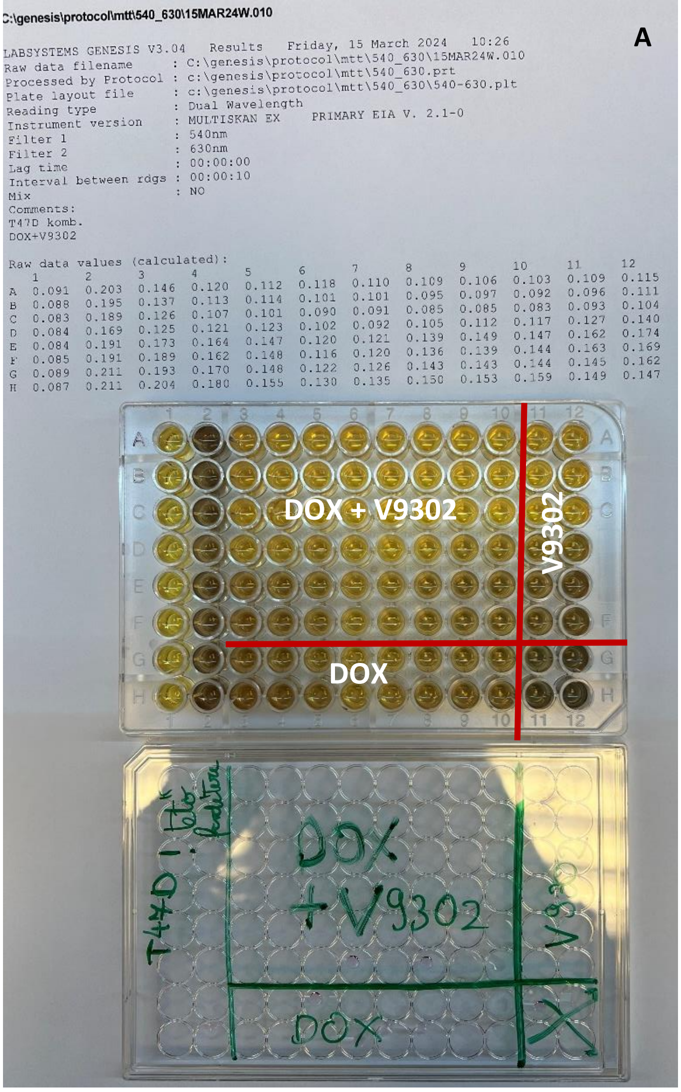

	1	2	3	4	5	6	7	8	9	10	11	12
A	0.091	0.203	0.146	0.120	0.112	0.118	0.110	0.109	0.106	0.103	0.109	0.115
B	0.088	0.195	0.137	0.113	0.114	0.101	0.101	0.095	0.097	0.092	0.096	0.111
C	0.083	0.189	0.126	0.107	0.101	0.090	0.091	0.085	0.085	0.083	0.093	0.104
D	0.084	0.169	0.125	0.121	0.123	0.102	0.092	0.105	0.112	0.117	0.127	0.140
E	0.084	0.191	0.173	0.164	0.147	0.120	0.121	0.139	0.149	0.147	0.162	0.174
F	0.085	0.191	0.189	0.162	0.148	0.116	0.120	0.136	0.139	0.144	0.163	0.169
G	0.089	0.211	0.193	0.170	0.148	0.122	0.126	0.143	0.143	0.144	0.145	0.162
H	0.087	0.211	0.204	0.180	0.155	0.130	0.135	0.150	0.153	0.159	0.149	0.147

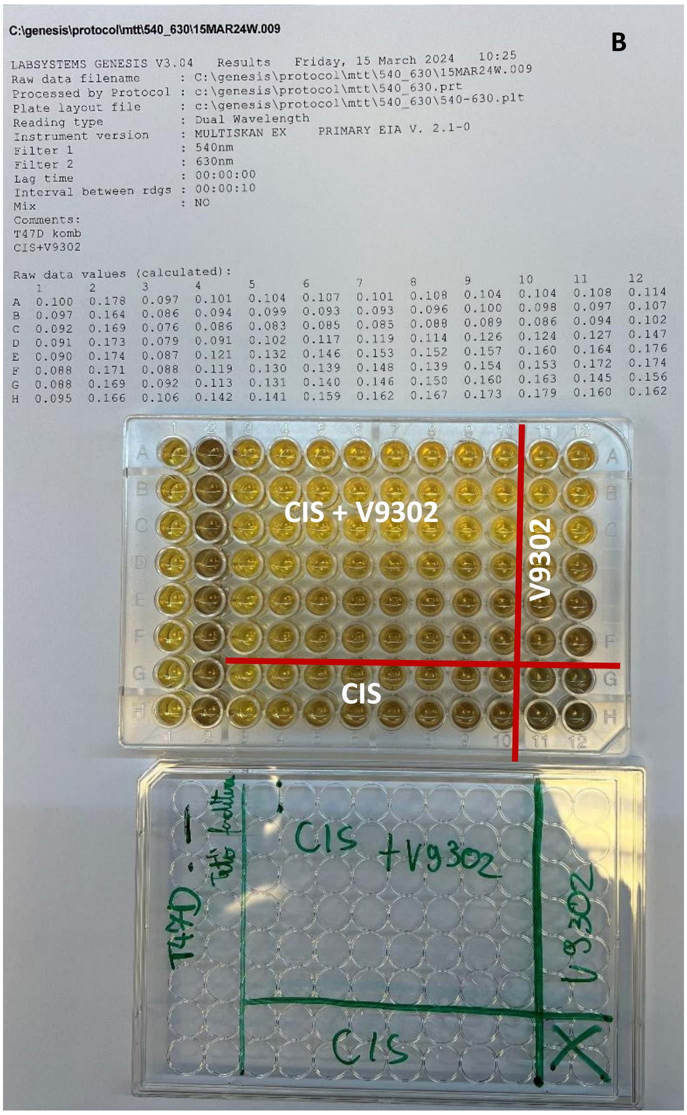

	1	2	3	4	5	6	7	8	9	10	11	12
A	0.100	0.178	0.097	0.101	0.104	0.107	0.101	0.108	0.104	0.104	0.108	0.114
B	0.097	0.164	0.086	0.094	0.099	0.093	0.093	0.096	0.100	0.098	0.097	0.107
C	0.092	0.169	0.076	0.086	0.083	0.085	0.085	0.088	0.089	0.086	0.094	0.102
D	0.091	0.173	0.079	0.091	0.102	0.117	0.119	0.114	0.126	0.124	0.127	0.147
E	0.090	0.174	0.087	0.121	0.132	0.146	0.153	0.152	0.157	0.160	0.164	0.176
F	0.088	0.171	0.088	0.119	0.130	0.139	0.148	0.139	0.154	0.153	0.172	0.174
G	0.088	0.169	0.092	0.113	0.131	0.140	0.146	0.150	0.160	0.163	0.145	0.156
H	0.095	0.166	0.106	0.142	0.141	0.159	0.162	0.167	0.173	0.179	0.160	0.162

Figure S2A: Photo of the combination plate of doxorubicin and V9302 on T47D cells. **B:** Photo of the combination plate of cisplatin and V9302 on T47D cells.

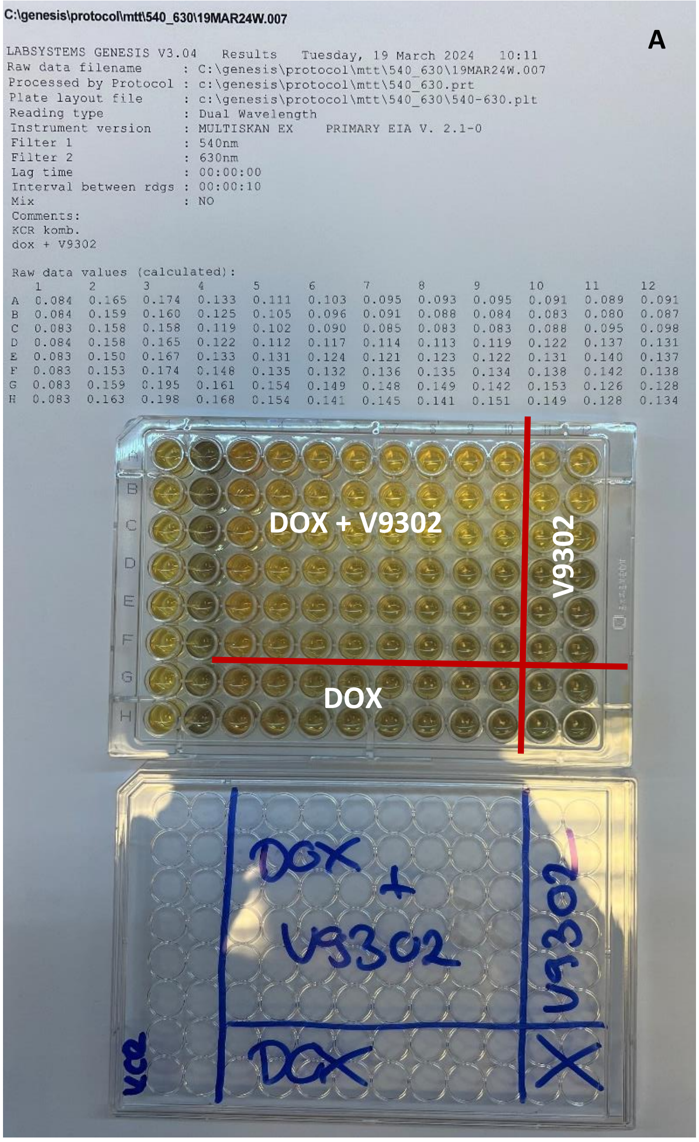

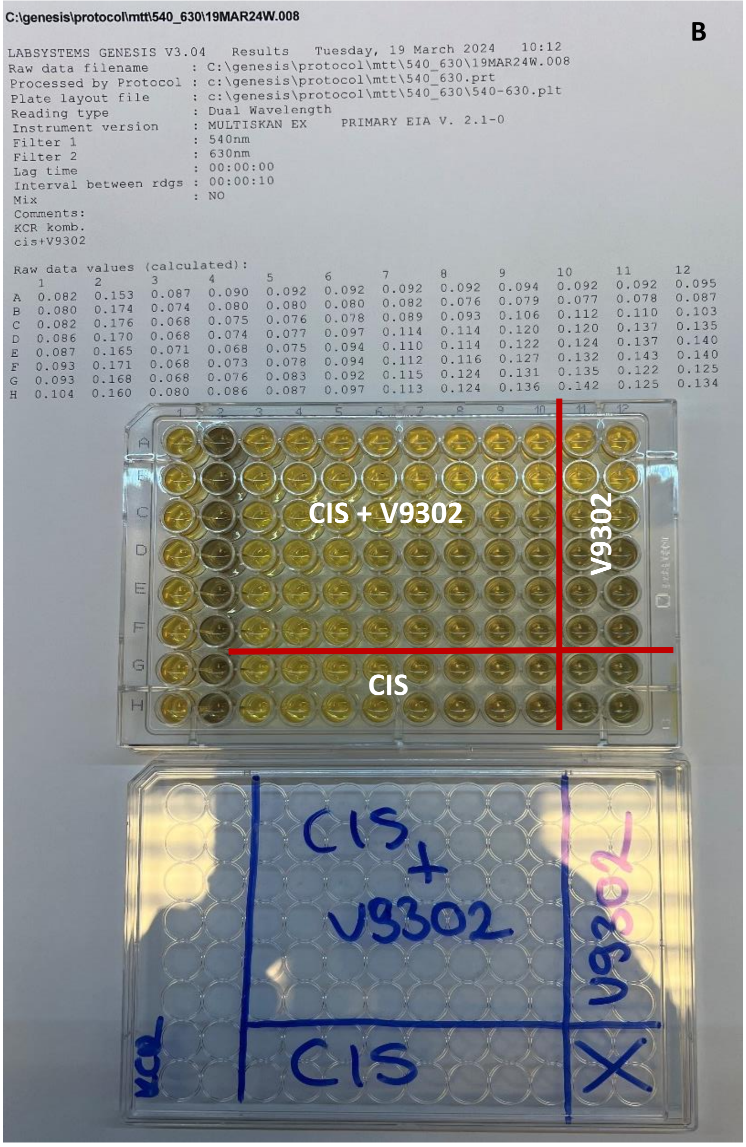

Figure S3A: Photo of the combination plate of doxorubicin and V9302 on KCR cells. **B:** Photo of the combination plate of cisplatin and V9302 on KCR cells.

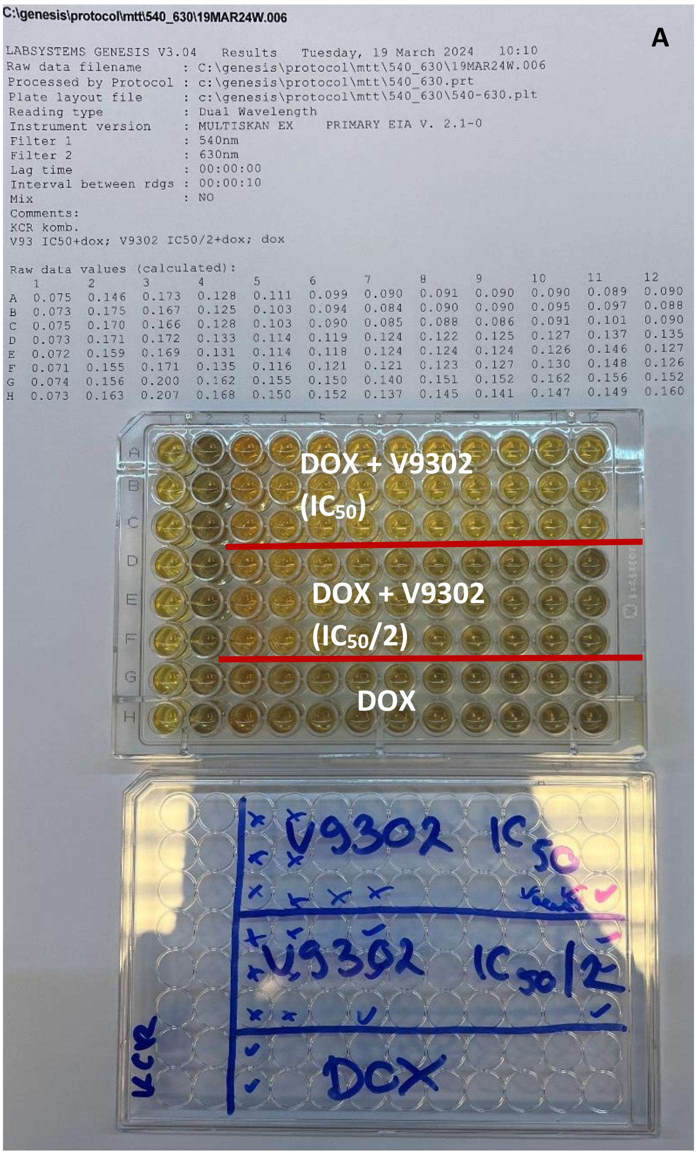
C:\genesis\protocol\mtt\540_630\19MAR24W.006

LABSYSTEMS GENESIS V3.04 Results Tuesday, 19 March 2024 10:10
Raw data filename : C:\genesis\protocol\mtt\540_630\19MAR24W.006
Processed by Protocol : c:\genesis\protocol\mtt\540_630.prt
Plate layout file : c:\genesis\protocol\mtt\540_630\540-630.plt
Reading type : Dual Wavelength
Instrument version : MULTISKAN EX PRIMARY EIA V. 2.1-0
Filter 1 : 540nm
Filter 2 : 630nm
Lag time : 00:00:00
Interval between rdgs : 00:00:10
Mix : NO
Comments:
KCR komb.
V93 IC50+dox; V9302 IC50/2+dox; dox

Raw data values (calculated):

	1	2	3	4	5	6	7	8	9	10	11	12
A	0.075	0.146	0.173	0.128	0.111	0.099	0.090	0.091	0.090	0.090	0.089	0.090
B	0.073	0.175	0.167	0.125	0.103	0.094	0.084	0.090	0.090	0.095	0.097	0.088
C	0.075	0.170	0.166	0.128	0.103	0.090	0.085	0.088	0.086	0.091	0.101	0.090
D	0.073	0.171	0.172	0.133	0.114	0.119	0.124	0.122	0.125	0.127	0.137	0.135
E	0.072	0.159	0.169	0.131	0.114	0.118	0.124	0.124	0.124	0.126	0.146	0.127
F	0.071	0.155	0.171	0.135	0.116	0.121	0.121	0.123	0.127	0.130	0.148	0.126
G	0.074	0.156	0.200	0.162	0.155	0.150	0.140	0.151	0.152	0.162	0.156	0.152
H	0.073	0.163	0.207	0.168	0.150	0.152	0.137	0.145	0.141	0.147	0.149	0.160

Figure S4A: Photo of the combination plate of doxorubicin and V9302 (at concentration IC_{50} and $IC_{50}/2$ on KCR cells.

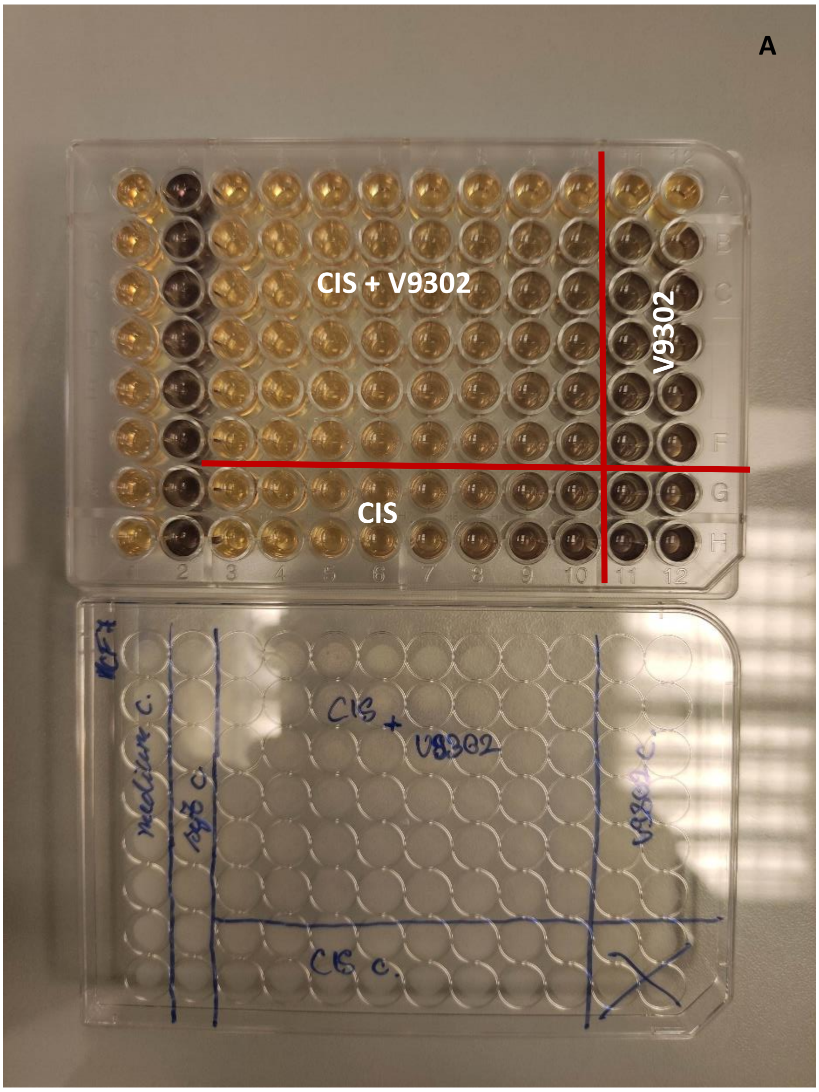

Figure S5A: Photo of the combination plate of cisplatin and on MCF-7 cells.

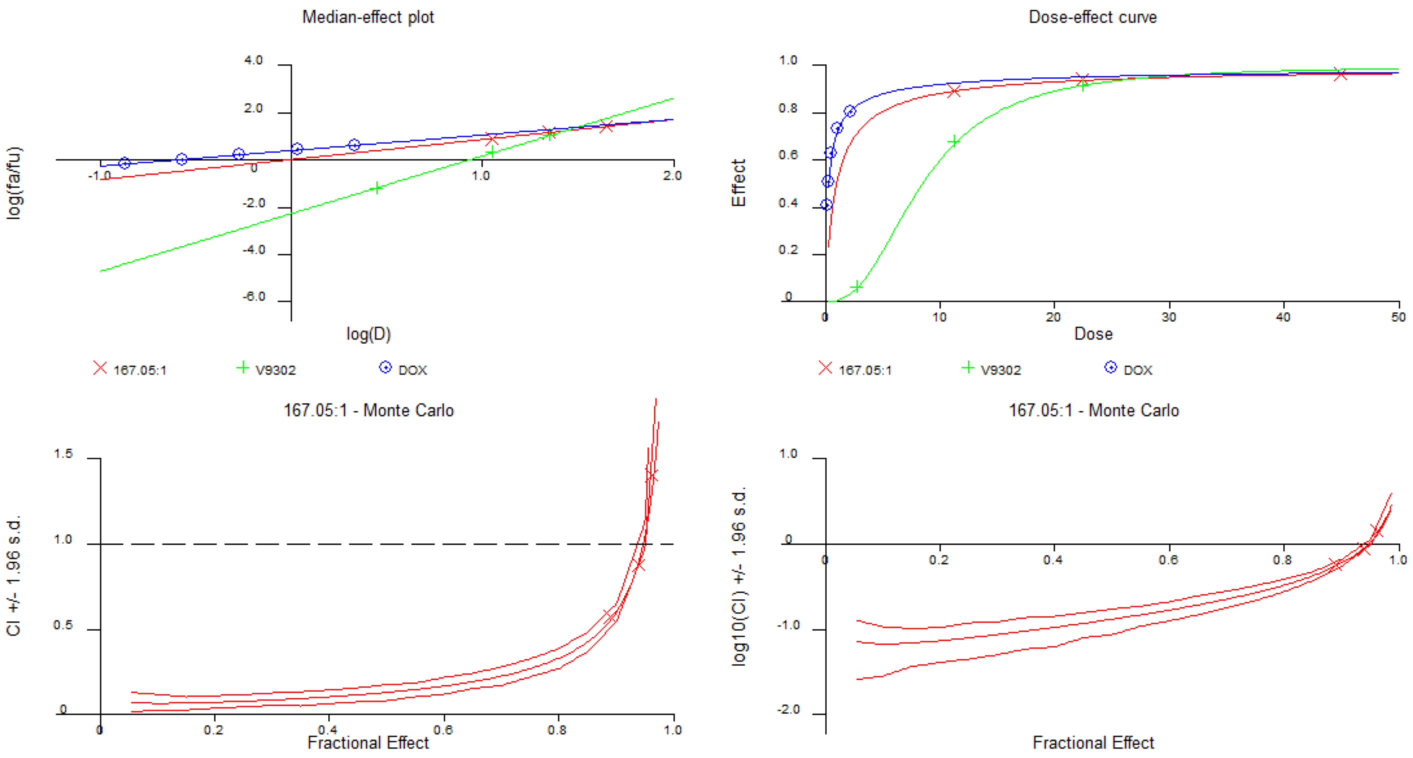

Figure S6. Graphs of checkerboard combination assay on MDA-MB-231 cells with doxorubicin and V9302 at ratio 167.05:1.

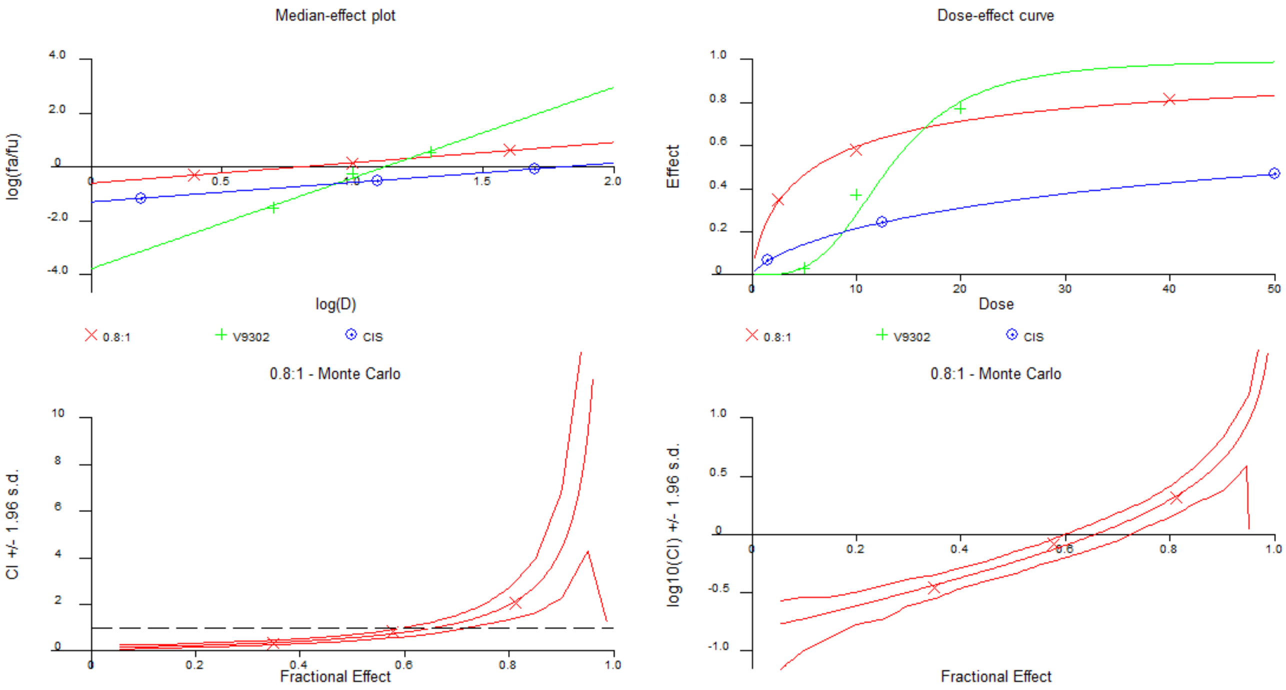

Figure S7. Graphs of checkerboard combination assay on MDA-MB-231 cells with cisplatin and V9302 at ratio 0.9:1.

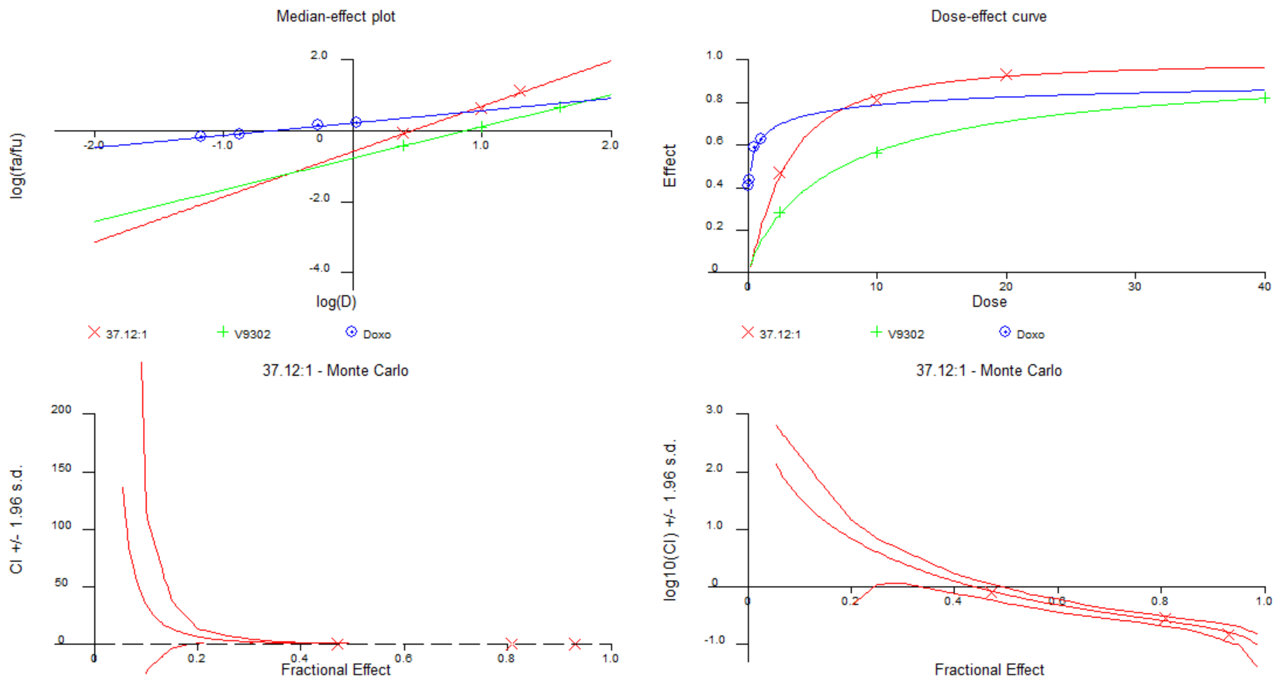

Figure S8. Graphs of checkerboard combination assay on T47D cells with doxorubicin and V9302 at ratio 37.12:1.

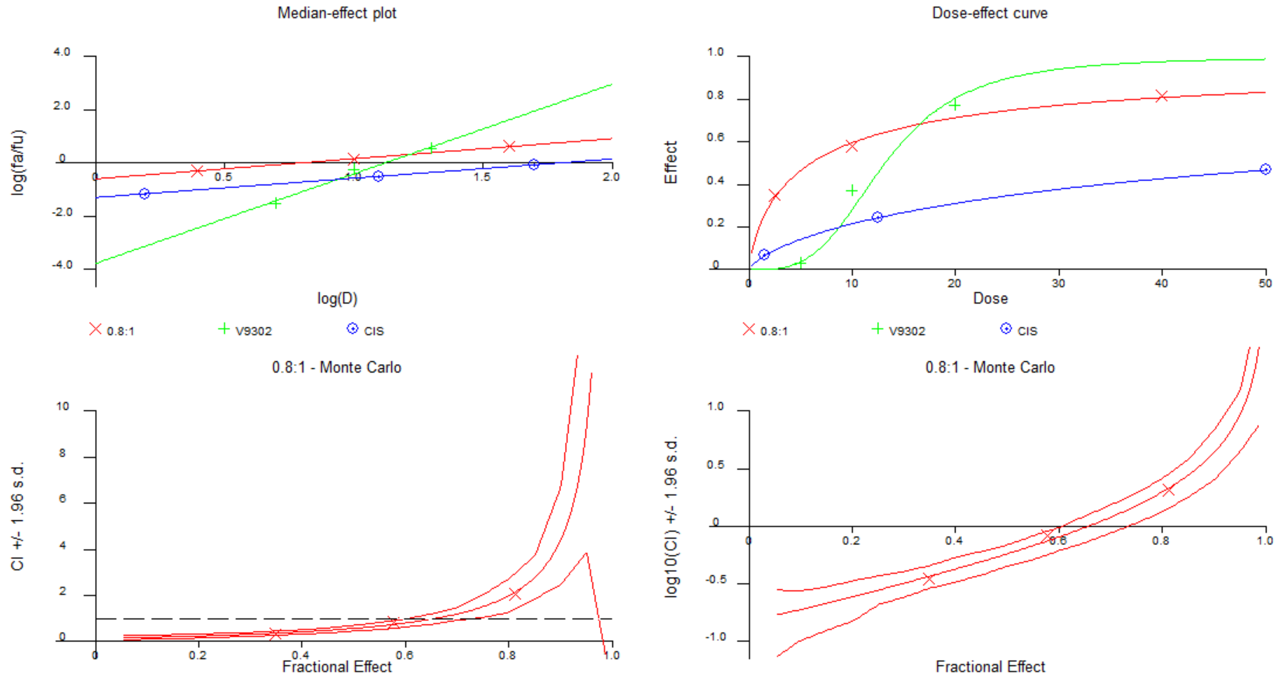

Figure S9. Graphs of checkerboard combination assay on T47D cells with cisplatin and V9302 at ratio 0.8:1.

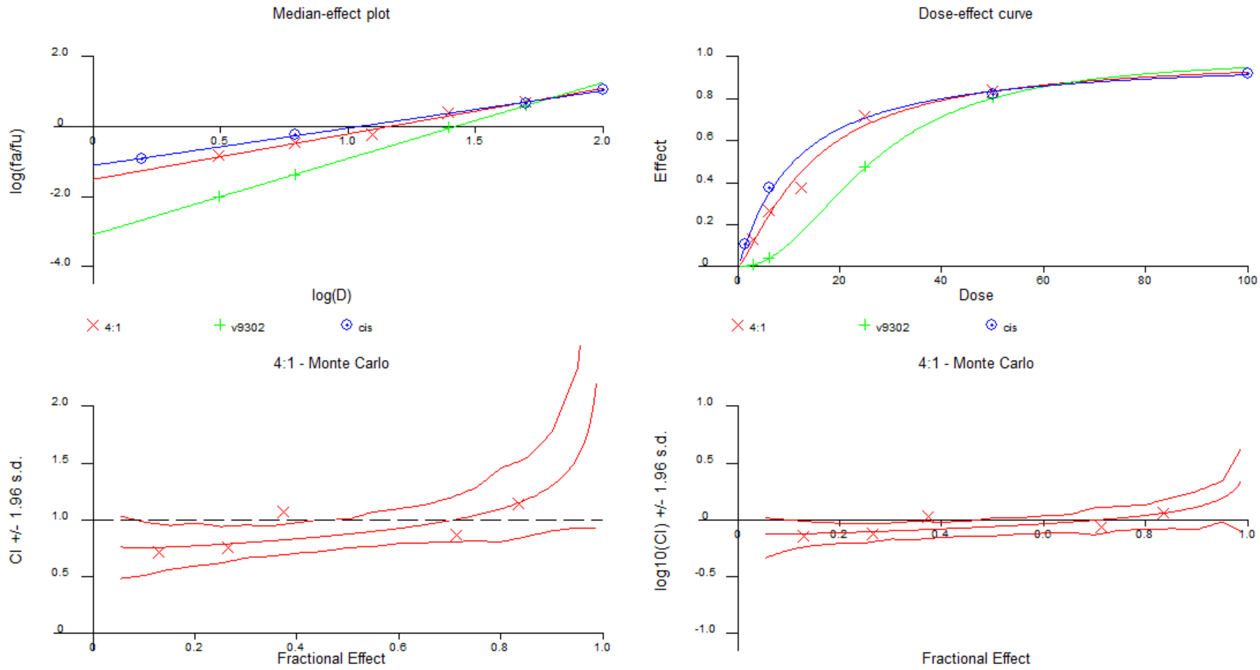

Figure S10. Graphs of checkerboard combination assay on KCR cells with cisplatin and V9302 at ratio 4:1.

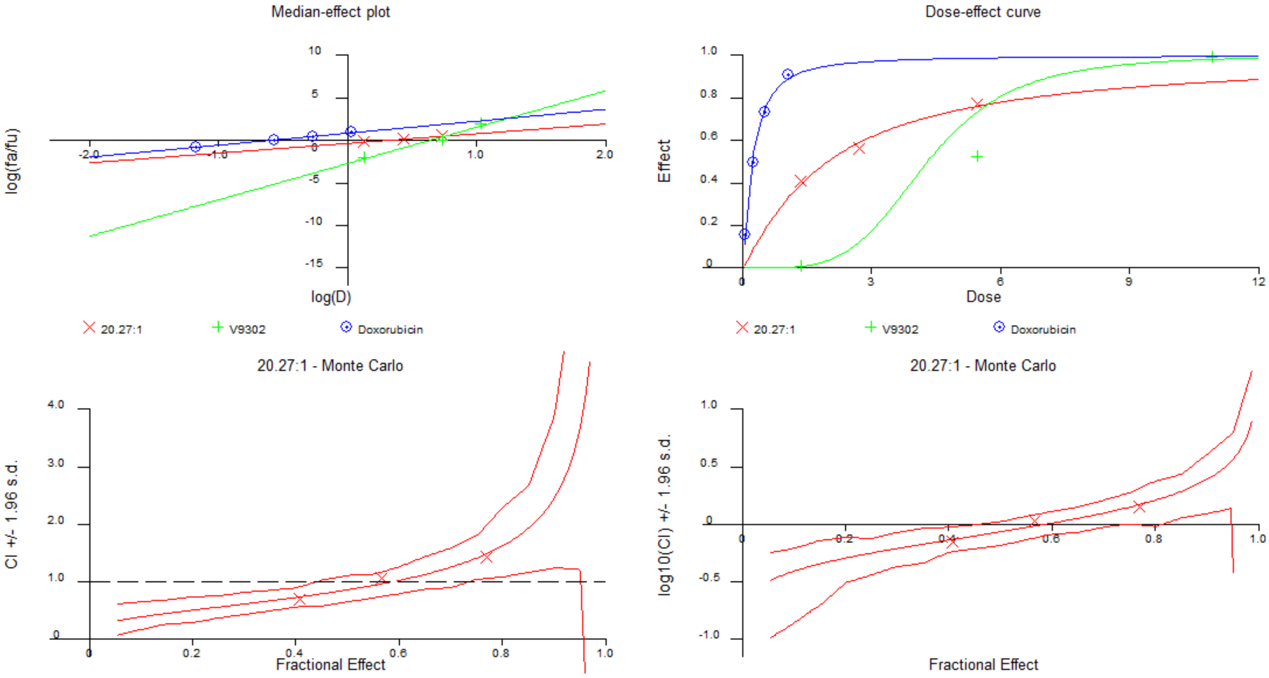

Figure S11. Graphs of checkerboard combination assay on MCF-7 cells with doxorubicin and V9302 at ratio 20.27:1.

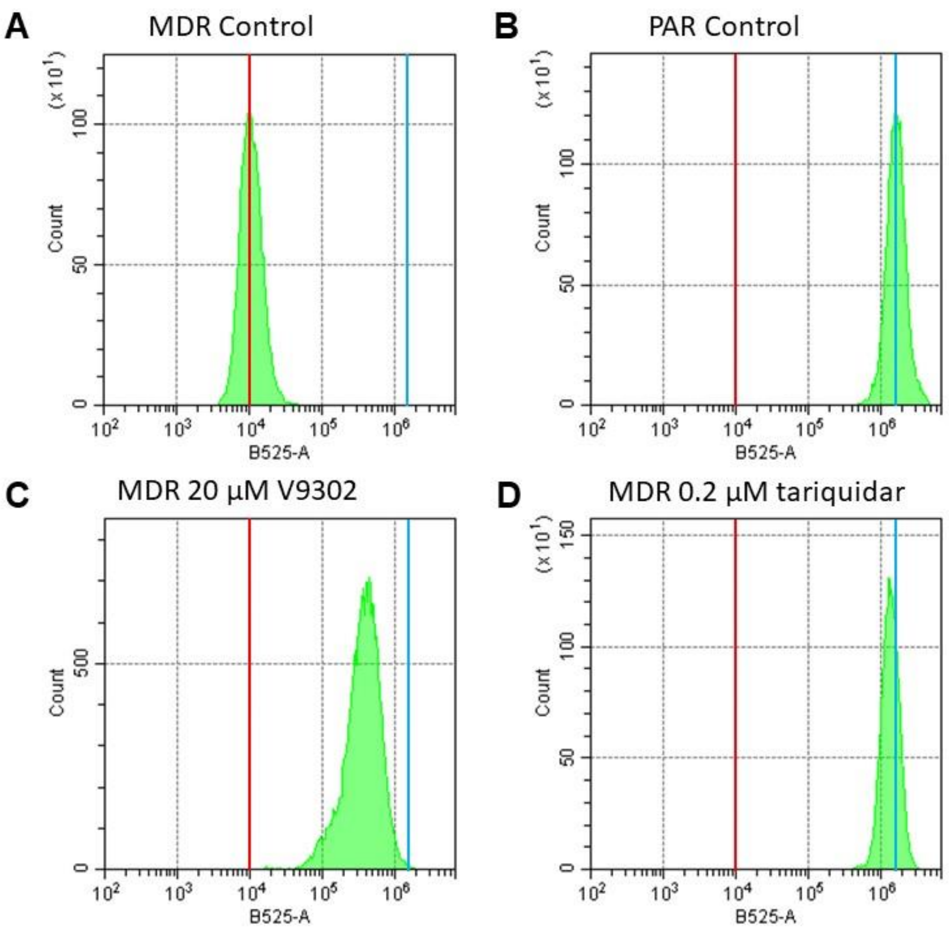

Figure S12. Rhodamine-123 accumulation by parental (PAR) L5178Y mouse T-lymphoma and its Pgp overexpressing counterpart (MDR). Tariquidar was used at 0.2 µM as a positive control on MDR cells, V9302 was applied at 20 µM on MDR cells.
